# Reformulation of a crystalline amino acid reference diet improves oxidation response but remains insufficient for assessing methionine bioavailability in extruded dog diets

**DOI:** 10.1093/jas/skaf364

**Published:** 2025-10-22

**Authors:** Michelina Crosbie, James R Templeman, Julia G Pezzali, Alexandra Rankovic, Glenda Courtney-Martin, Crystal Levesque, Leslie Hancock-Monroe, Preston R Buff, Daniel A Columbus, Anna K Shoveller

**Affiliations:** Department of Animal Biosciences, University of Guelph, Guelph, ON, N1G 2W1, Canada; Department of Animal Biosciences, University of Guelph, Guelph, ON, N1G 2W1, Canada; Department of Animal Biosciences, University of Guelph, Guelph, ON, N1G 2W1, Canada; Department of Animal Biosciences, University of Guelph, Guelph, ON, N1G 2W1, Canada; Department of Nutritional Sciences, University of Toronto, Toronto, ON, M5S 1A8, Canada; Department of Animal Sciences, South Dakota State University, Brookings, SD, 57007, United states; The J.M. Smucker Co, Orrville, OH 44667-0280, United states; The J.M. Smucker Co, Orrville, OH 44667-0280, United states; Department of Animal and Poultry Science, University of Saskatchewan, Saskatoon, SK S7N 5A8, Canada; Department of Animal Biosciences, University of Guelph, Guelph, ON, N1G 2W1, Canada

**Keywords:** canine nutrition, chicken meal, indicator amino acid oxidation, metabolic availability, peas

## Abstract

Previously, a semi-synthetic crystalline amino acid (AA) reference (BAS) diet, limited in methionine (Met) but providing indispensable AAs (IDAA) at 120% of Association of American Feed Control Officials (AAFCO) recommendations for adult dogs had no oxidation response, indicating another limiting AA. Thus, we could not determine the metabolic availability (MA) of dietary Met in peas and chicken meal (ChM), the latter having the lowest oxidation level. Therefore, we sought to determine if reformulating the BAS diet AA profile to match the IDAA content of the ChM diet would allow for determination of the MA of Met in peas and ChM over two experiments. In the reformulated BAS63+ diet, we increased arginine, histidine, isoleucine, leucine, lysine, threonine, and valine relative to the original BAS63− diet, while maintaining Met at 63% of requirement (0.33% DM). In Exp 1, one neutered male dog (3 years old; 30.3 kg BW) was used in a two-period switchback design to receive BAS63− and BAS63+. Following a 2-d adaptation, the indicator AA oxidation (IAAO) technique was used. The dog received 13 small meals, with meal 6 containing a priming dose (9.4 mg/kg BW) of L-[1-^13^C]-phenylalanine (Phe, 99%), followed by a constant dose (2.4 mg/kg BW) in meals 6–13. Breath samples were collected to measure ^13^CO_2_ enrichment, and oxidation was calculated (F^13^CO_2_/kg BW/h). The BAS63+ diet improved oxidation by 84% and was used in Exp 2. In Exp 2, seven neutered male mixed-breed dogs (3 years old; 26.3 ± 2.0 kg BW) were used in a partially replicated 5 × 5 Latin square design to receive isonitrogenous and isoenergetic dietary treatments: BAS63+, ChM63 (ChM and lamb-based diet), and PEA63 (green pea and lamb-based diet), all providing Met at 63% (0.33% DM) of requirement. Two additional BAS diets were created with Met at 19% (BAS19+; 0.10% DM) and 40% (BAS40+; 0.21% DM) of requirement. This created two and three graded levels of Met for ChM and peas, and the BAS diet, respectively, allowing for a slope ratio approach to quantify MA using the BAS19+ diet as the common first point. The IAAO technique was performed in Exp 2 using the same protocol. Data were analyzed using Proc GLIMMIX with dog and period as random effects and diet, %Met, and their interaction as fixed effects. Overall, ChM had the lowest oxidation level, and the MA of Met in peas was 58% relative to ChM. The higher oxidation in the BAS63+ compared to ChM suggests another AA may still be limiting in the BAS diet.

## Teaser

Reformulating a crystalline amino acid (AA) reference diet by increasing seven indispensable AAs to match the profile of a chicken meal diet improved protein synthesis by 84%. However, chicken meal still supported lower oxidation than the reformulated diet, and methionine availability in peas was only 58% that of chicken meal.

## Introduction

When one amino acid (AA) is limiting in the diet, whole-body protein synthesis will be limited, and all other AA provided in excess will be catabolized or oxidized (Moehn et al., 2005). Therefore, the rate of oxidation is inversely proportional to the rate of protein synthesis (Moehn et al., 2005). The indicator AA oxidation (IAAO) technique allows for the measurement of this oxidation response via the use of a ^13^C-labelled AA and can be used to determine the metabolic availability (MA) of an AA in an ingredient (Moehn et al., 2005). MA of an AA is the proportion of that AA in an ingredient that is not only digestible but is also available for protein synthesis (Moehn et al., 2005). Determining the MA of an AA in an ingredient using the IAAO technique requires that diets be formulated to contain at least three levels of the AA of interest, and that the AA of interest be provided below the requirement (Moehn et al., 2005). Additionally, diets must be isonitrogenous and isoenergetic and all other indispensable and related AA (i.e., cysteine [Cys] in the case of methionine [Met]) must be provided in excess of the requirement to allow for a slope ratio approach to be used (Moehn et al., 2005). The MA of AA in ingredients can then be calculated in relation to a reference protein considered to have 100% MA of AA (Moehn et al., 2005).

Both peas and chicken meal (ChM) are commonly used plant and animal protein sources in extruded dog foods (Plantz, 2017; Faber et al., 2010; Mansilla et al., 2019). Particularly, peas are known to contain low concentrations of sulfur AA and have a low Met:Cys (Met:Cys = ≤1:1; NRC, 2006; Olsen et al., 2020). Previous work in our laboratory (Crosbie et al., 2025) attempted to determine the MA of Met in both ChM and peas using a reference diet containing crystalline AA formulated to 120% of the American Association of Feed Control Officials (AAFCO) recommendations for adult dogs at maintenance (AAFCO, 2023). This AA provision was approximately 143% of the National Research Council (NRC) recommended allowance for adult dogs at maintenance (NRC, 2006). However, this reference diet AA formulation did not produce an IAAO response, indicating that another AA or total nitrogen in the reference diet was limiting and driving protein synthesis (Moehn et al., 2005; Crosbie et al., 2025). Thus, we were unable to determine the MA of Met in both ChM and peas, as this requires comparison to a reference diet. However, we determined that the Met in ChM produced the greatest (ie most negative) oxidation response (Crosbie et al., 2025). All diets were isonitrogenous in said study, however, the ChM diet provided on average 20% more indispensable AA (IDAA) than the reference diet used, suggesting further that another IDAA (other than Met) was limiting in our crystalline AA reference diet (Crosbie et al., 2025). Therefore, the objectives of the current study were twofold; first, to determine if reformulating a crystalline AA reference diet to match our previously used ChM diet would improve the oxidation response compared to our previous work in a pilot study; and second, to determine if this reformulated reference diet would allow us to calculate the MA of Met in both peas and ChM in a full study. We hypothesize that reformulating the crystalline AA reference diet to match a ChM-based diet, previously determined to have the greatest oxidation response, would also result in a greater oxidation response and allow us to determine the MA of Met in both peas and ChM.

## Materials and Methods

The experimental protocol and study design were reviewed and approved by the University of Guelph Animal Care Committee (AUP# 4531). Handling and care of the animals was in accordance with the Canadian Council on Animal Care Guidelines (CCAC, 2020).

### Animals, housing, and acclimation to oxidation chambers

This study utilized seven neutered male mixed-breed hound dogs, averaging 26.3 ± 2.0 kg in body weight (BW) and each 3 yr old. The dogs were originally sourced from Marshall Bioresources (Waverly, NY, United States of America) and housed at the Central Animal Facility at the University of Guelph (Guelph, ON, Canada). Each dog was individually housed in kennels measuring 3.7 m in length, 2.0 m in width, and 2.0 m in height. The kennels allowed for both nose-to-nose interaction and visual contact with neighboring dogs. Environmental conditions within the kennel room were kept constant, with an average room temperature of 20.8°C, relative humidity maintained of 59.0%, and a 12:12 h light:dark cycle. Dogs had continuous access to water and nylon and rubber toys in their kennels. Dogs received 20 min of supervised outdoor walks 6 d/wk; when weather conditions were unsuitable, walks were conducted indoors. All dogs had previously been adapted to eating multiple meals in crates, as detailed by Shoveller et al. (2017) and Crosbie et al. (2024).

### Experiment 1: Pilot trial to determine reformulation of a crystalline amino acid reference diet

#### Diets and study design

One of the dogs (3 yr old; 30.3 kg BW) was used to complete this pilot trial. One extruded kibble diet was formulated to act as a reference diet to determine the MA of Met in peas and ChM (BAS; Table 1). This diet was a corn starch and barley-based extruded diet providing Met at 19% of its requirement for Labrador Retrievers (0.10% dry matter [DM], Table 3; Mansilla et al., 2020) and was formulated to contain a calculated metabolizable energy (ME) of 3500 kcal/kg as-fed (Table 2) using the modified Atwater equation. Barley was used to improve the extrusion of a primarily corn-starch-based diet and its inherently low Met content, which allowed for changes in the MA of Met to be reflective of the inclusion level of crystalline AA (Moehn et al., 2005). Inclusion levels of vitamins and minerals provided in the BAS diet were based on the levels of vitamins and minerals provided by the primary ingredients (i.e., barley) to provide all vitamins and minerals above the AAFCO recommendations for adult dogs at maintenance (AAFCO, 2023). Processing parameters during extrusion of this diet achieved a specific mechanical energy (HP/hr/ton) off the extruder of 95.3.

**Table 1.** Ingredient composition of basal (BAS19), chicken (ChM63) and pea (PEA63) treatment diets used to determine the metabolic availability of Met in peas and chicken meal on as-fed (%) basis^1^

	Diet		
Ingredient, %	BAS19	ChM63	PEA63
**Green peas, chipped**	–	–	37.89
**Chicken meal**	–	6.58	–
**Lamb meal**	–	15.00	15.00
**Lamb, deboned**	–	9.50	9.50
**Corn starch**	44.88	29.96	–
**Barley, pearled**	25.82	25.00	25.00
**Chicken fat**	13.52	6.87	6.96
**Beet pulp**	3.00	3.00	2.5
**Animal digest**	1.00	1.00	1.00
**Potassium chloride**	1.50	0.80	0.11
**Salt**	1.00	1.00	0.50
**Vitamin mix**	0.32	0.30	0.26
**Mineral mix**	0.50	0.30	0.21
**Choline chloride**	0.40	0.15	0.12
**Naturox^2^, dry**	0.03	0.03	0.03
**Naturox^2^, liquid**	0.025	0.025	0.025

1 Formulation of all three test diets are as formulated and do not reflect any blending of diets. BAS19 = Corn starch and barley-based reference diet; ChM63 = Chicken meal diet containing chicken meal and lamb as the primary protein sources; PEA63 = Pea diet containing peas and lamb as the primary protein sources.

2 Naturox: a commercial natural antioxidant blend containing mixed tocopherols, manufactured by Kemin Industries (Des Moines, IA, United States).

**Table 2.** Analyzed and calculated nutrient and amino acid contents of a basal (BAS63−) reference diet with 63% of the Met requirement and all indispensable amino acids provided at 120% of AAFCO recommendations for adult dogs at maintenance, and a BAS63+ reference diet with 63% of the Met requirement and all indispensable amino acids matched to the chicken (ChM63) diet after blending and supplementation on DM-basis (unless specified)

	Diet					
Item	BAS63−		BAS63+		ChM63^1^	
**% Met req.^2^**	63		63		63	
**ME, kcal/kg as-fed^3^**	3403		3403		3555	
**Dry Matter, %**	88.30		88.30		93.50	
**Crude Protein, %^4^**	23.39 (5.03)		23.39 (5.03)		23.39 (17.54)	
**Crude Fat, %**	15.40		15.40		13.69	
**Crude Fiber, %**	1.40		1.40		2.04	
**Total dietary fiber, % as-fed**	5.93		5.93		8.30	
**Soluble dietary fiber, % as-fed**	1.45		1.45		2.39	
**Insoluble dietary fiber, % as-fed**	4.48		4.48		5.91	
**NFE, g/100g as-fed^5^**	59.77		59.77		54.09	
**Ash, %**	9.3		9.3		8.3	
**Indispensable AA, %**	Diet^6^	Free^7^	Diet	Free	Diet	Free
** Arg**	0.24	0.38	0.24	0.88	1.12	
** His**	0.10	0.13	0.10	0.24	0.34	
** Ile**	0.18	0.28	0.18	0.41	0.59	
** Leu**	0.34	0.48	0.34	0.84	1.18	
** Lys**	0.17	0.59	0.17	0.69	0.86	
** Met**	0.10	0.23	0.10	0.23	0.32	
** Phe**	0.24	0.77	0.24	0.77	0.69	0.32
** Thr**	0.17	0.41	0.17	0.46	0.63	
** Trp**	0.06	0.13	0.06	0.13	0.16	0.03
** Val**	0.24	0.35	0.24	0.54	0.78	
**Dispensable AA, %**	Diet	Free	Diet	Free	Diet	Free
** Ala**	0.22	14.29	0.22	12.47	1.07	5.24
** Asp**	0.31		0.31		1.29	
** Cystine^8^**	0.11	0.29	0.11	0.29	0.22	0.18
** Tyr**	0.18	0.93	0.18	0.93	0.53	0.58
** Glu**	0.95		0.95		2.56	
** Gly**	0.22		0.22		1.56	
** Pro**	0.47		0.47		1.36	
** Ser**	0.20		0.20		0.72	

1 Nutrient composition after blending and supplementation of the ChM63 diet from Crosbie et al. (2025) was not tested in this experiment and is provided here for comparison only.

2 Methionine requirement determined as Met requirement for Labrador Retrievers (0.52 g/100g DM; Mansilla et al., 2020).

3 ME calculated using modified Atwater calculation.

4 Presented as: Final Crude Protein content after supplementation with Ala (Crude Protein content of the diet prior to supplementation with Ala).

5 NFE calculated using NFE, g/100g = 100 – (moisture + Crude Protein + Crude Fat + Crude Fiber + Ash).

6 The amount of AA (% DM) that is found in the diet as determined by analytical methods.

7 The amount of AA (% DM) added as free-AA to ensure the provision of all indispensable AA in the BAS63− diet (except for Met) was at least 120% of the AAFCO recommendation for adult dogs at maintenance (AAFCO, 2023) and BAS63+ diets (except for Met) were provided to match the ChM63 diet which had the greatest oxidation response (F^13^CO_2_) previously (Crosbie et al., 2025). The provision of Tyr and Phe across all treatments was matched to the PEA63 diet used in Crosbie et al. (2025) via free-AA supplementation and Cys was supplemented as free-AA to achieve a provision of 0.40 g/100g DM across all diets. In the ChM63 diet Trp was supplemented to ensure its provision was at least 120% of the AAFCO recommendation for adult dogs at maintenance (AFFCO, 2023). For Ala, this represents the amount of AA (% DM) added to the diet in order to make diets isonitrogenous and match to the crude protein in the PEA63 diet.

8 Values reported are on a cystine-basis. However, cysteine was used as the free-AA as it was easier to work with in solution. Cysteine dosage was determined relative to the mol/kg of cystine provided in the diet.

**Table 3.** Analyzed and calculated nutrient and amino acid contents of the basal (BAS19) reference diet with 19% of the Met requirement, BAS40 diet with 40% of the Met requirement, and the BAS63, chicken (ChM63), and pea (PEA63) diets with 63% of the Met requirement after blending and supplementation used to determine the metabolic availability of Met in peas and chicken meal on DM-basis (unless specified)

	Diet									
Item	BAS19+		BAS40+		BAS63+		ChM63		PEA63	
**% Met req.^1^**	19		40		63		63		63	
**ME, kcal/kg as-fed^2^**	3403		3403		3403		3555		3574	
**Dry matter, %**	88.30		88.30		88.30		93.50		93.34	
**Crude protein, %^3^**	23.39 (5.03)		23.39 (5.03)		23.39 (5.03)		23.39 (17.54)		23.39	
**Crude fat, %**	15.40		15.40		15.40		13.69		13.39	
**Crude fiber, %**	1.40		1.40		1.40		2.04		2.16	
**Total dietary fiber, % as-fed**	5.93		5.93		5.93		8.30		12.80	
**Soluble dietary fiber, % as-fed**	1.45		1.45		1.45		2.39		3.15	
**Insoluble dietary fiber, % as-fed**	4.48		4.48		4.48		5.91		9.60	
**NFE, g/100g as-fed^4^**	59.77		59.77		59.77		54.09		50.17	
**Ash, %**	9.3		9.3		9.3		8.3		7.0	
**Indispensable AA, %**	Diet^5^	Free^6^	Diet	Free	Diet	Free	Diet	Free	Diet	Free
** Arg**	0.24	0.88	0.24	0.88	0.24	0.88	1.12		1.76	
** His**	0.10	0.24	0.10	0.24	0.10	0.24	0.34		0.49	
** Ile**	0.18	0.41	0.18	0.41	0.18	0.41	0.59		0.84	
** Leu**	0.34	0.84	0.34	0.84	0.34	0.84	1.18		1.59	
** Lys**	0.17	0.69	0.17	0.69	0.17	0.69	0.86		1.27	
** Met**	0.10		0.10	0.11	0.10	0.23	0.32		0.33	
** Phe**	0.24	0.77	0.24	0.77	0.24	0.77	0.69	0.32	1.01	
** Thr**	0.17	0.46	0.17	0.46	0.17	0.46	0.63		0.84	
** Trp**	0.06	0.13	0.06	0.13	0.06	0.13	0.16	0.03	0.21	
** Val**	0.24	0.54	0.24	0.54	0.24	0.54	0.78		1.04	
**Dispensable AA, %**	Diet^7^	Free^8^	Diet	Free	Diet	Free	Diet	Free	Diet	Free
** Ala**	0.22	12.61	0.22	12.54	0.22	12.47	1.07	5.24	1.21	
** Asp**	0.31		0.31		0.31		1.29		2.02	
** Cystine^7^**	0.11	0.29	0.11	0.29	0.11	0.29	0.22	0.18	0.30	0.10
** Tyr**	0.18	0.93	0.18	0.93	0.18	0.93	0.53	0.58	0.74	0.37
** Glu**	0.95		0.95		0.95		2.56		3.58	
** Gly**	0.22		0.22		0.22		1.56		1.55	
** Pro**	0.47		0.47		0.47		1.36		1.49	
** Ser**	0.20		0.20		0.20		0.72		0.98	

1 Methionine requirement determined as Met requirement for Labrador Retrievers (0.52 g/100g DM; Mansilla et al., 2020).

2 ME calculated using modified Atwater calculation.

3 Presented as: Final Crude Protein content after supplementation with Ala (Crude Protein content of the diet prior to supplementation with Ala).

4 NFE calculated using NFE, g/100g = 100 – (moisture + Crude Protein + Crude Fat + Crude Fiber + Ash).

5 The amount of AA (% DM) that is found in the diet as determined by analytical methods.

6 The amount of AA (% DM) added as free-AA to ensure the provision of all indispensable AA in the BAS19, BAS40, and BAS63 diets (except for Met) were provided to match the ChM63 diet except for the provision of Tyr, and Phe across all treatments, which was matched to the PEA63 diet via free-AA supplementation. Cysteine was supplemented as free-AA to achieve a provision of 0.40 g/100g DM across all diets. In the ChM63 diet Trp was supplemented to ensure its provision was at least 120% of the AAFCO recommendation for adult dogs at maintenance (AFFCO, 2023). For Ala, this represents the amount of AA (% DM) added to the diet in order to make diets isonitrogenous and match to the crude protein in the PEA63 diet.

7 Values reported are on a cystine-basis. However, cysteine was used as the free-AA as it was easier to work with in solution. Cysteine dosage was determined relative to the mol/kg of cystine provided in the diet.

Two BAS diet treatments (BAS63− and BAS63+) were created through supplementation of IDAA to the BAS diet which were added as free AA, on an equimolar basis, in a solution of distilled water heated to a maximum of 50°C to ensure free AA underwent minimal heat treatment and were completely dissolved (Table 2). These solutions were made in small batches and stored at 4°C for a maximum of 3 d to prevent precipitation of AA contents and top-dressed on the diet using a syringe before feeding and dosed as a proportion of the diet fed. As the BAS diet before supplementation contained barley, which provided some protein-bound AA, the addition of supplemental AA made this reference diet semi-synthetic. Both the BAS63− and BAS63+ diets provided Met at 63% of its requirement for Labrador Retrievers (0.52% DM; Mansilla et al., 2020). In the BAS63− diet, all IDAA were provided at 120% of the AAFCO recommendation for adult dogs at maintenance (AAFCO, 2023). Previous work in our laboratory (Crosbie et al., 2025) determined that formulating all IDAA, except for Met, to 120% of the AAFCO recommendations for adult dogs at maintenance did not produce an oxidation response (Moehn et al., 2005; AAFCO, 2023). In this previous work, we determined that the ChM diet produced the greatest oxidation response, therefore, to compare the response to oxidation we formulated a second dietary treatment (BAS63+) where all IDAA, except for Met, were provided to match the IDAA composition of the ChM diet used in this previous work (Table 2). To conduct an MA study using the IAAO technique, phenylalanine (Phe) was provided at 1.01% DM in both BAS diet treatments, which was greater than 200% of the AAFCO recommendation for adult dog at maintenance (1.01% versus 0.45% DM, respectively, Table 2; AAFCO, 2023). Tyrosine (Tyr) was supplied at 110% of the Phe provision on a DM-basis to prevent Phe from being shunted to Tyr synthesis, thereby promoting its use toward protein synthesis or oxidation (Shiman and Gray, 1998). Additionally, cysteine was maintained at 0.40% DM across all diets to prevent sulfur AA limitation when Met was provided at 63% of its requirement (0.33% DM versus 0.52% DM, respectively; Mansilla et al., 2020). This provision was over 60% of the AAFCO (2023) total sulfur AA recommendations on a DM basis (Table 2). The protein content of both treatments was matched to what had been used in our previous work (23.39% CP on a DM-basis; Crosbie et al. 2025) to ensure results were comparable. Both diets were supplemented with alanine (Ala) in solution to make diets isonitrogenous and prepared using the same standards outlined above.

The study used a two-period switchback design (*n* = 1), where the dog was randomly assigned to one of the two dietary treatments in each experimental period. During the 14-d diet adaptation period, the dog was fed a commercial wash-in diet (S6 Nutram Sound Balanced Wellness Adult Dog Food, chicken meal and brown rice recipe; Elmira Pet Products, Elmira, ON, Canada) once daily at 0800 h in amounts previously shown to maintain ideal individual BW based on historical feeding records. The dog was allowed 15 min to finish his daily meal. There were two 3-d experimental periods conducted consecutively; the first 2 d were diet adaptation to the treatment diet, followed by breath collection on day 3. During the 2-d diet adaptation period, IAAO studies typically require feeding animals the same amount of food in g/kg BW to ensure all dogs are adapted to receiving the same amount of dietary nitrogen (Moehn et al., 2005; Shoveller et al., 2017). However, previous work using the IAAO technique showed that, despite all dogs having similar ideal BW, their individual energy requirements varied. As a result, feeding all dogs the same amount would lead to weight loss in some and weight gain in others. Because weight loss and weight gain can alter metabolism, and maintaining ideal BW is a principle of MA IAAO studies, all dogs were fed to maintain ideal BW during the 2-d diet adaptation period (Moehn et al., 2005; Shoveller et al., 2017). Therefore, in this pilot study, the dog was also fed to maintain ideal BW during the 2-d adaptation period. To control for the variation introduced by individualized feeding, the same quantity of each experimental diet was fed on an energy basis within dog across all study days. Free AA were dosed as a proportion of the total diet to ensure consistent AA balance and intake relative to daily food amount. On IAAO breath collection days, food intake was restricted to 13 g/kg BW, representing 100% of the dog’s historical feeding allowance required to maintain ideal BW. This feeding protocol ensured complete consumption of test diets during IAAO and equivalent isotope delivery. This feeding protocol was repeated twice, allowing the dog to receive all treatments. Blood samples (5 mL) were collected within 30 min of the final meal on each IAAO day. Blood was collected from the cephalic vein in a 10 mL sodium heparin tube (Becton Dickinson Canada Inc., Mississauga, ON, Canada) and placed on ice. Once all samples were collected, 1 mL of whole blood was separated and stored at −80°C. The remaining 4 mL was centrifuged at 4 °C at 15,000×g for 15 min. Plasma was separated, and aliquots were stored at −80°C until analysis of fed-state plasma and whole blood AA concentrations.

#### Indicator amino acid oxidation study

Each IAAO study day was conducted according to Crosbie et al. (2024). The dog was first weighed to determine the appropriate feed amount, isotope dose, and AA solution volume. Following this, the dog was moved to an individual oxidation chamber to which it had previously been acclimated. In short, after 30 min of gas equilibration, triplicate resting volume of expired CO_2_ and O_2_ (VCO_2_; VO_2_; respectively) measurements were taken. Due to the increased variability associated with fed state-VCO_2_ in free-living animals, fasted state measurements were taken. The dog was then fed (time 0) their corresponding feed allowance divided into 13 equal small meals; where the first three meals were fed every 10 min to induce a fed state, and the other 10 meals were fed every 25 min. The total amount fed during the IAAO study was based on the BW measured that same morning after 22 h of fasting (13 g food/kg BW). Background ^13^C enrichment was determined by collecting CO_2_ samples over three consecutive 25 min periods. The sixth meal (95 min after first feeding) contained a priming dose of L-[1-^13^C]-Phe (9.4 mg/kg BW, 99%; Cambridge Isotope Laboratories, Inc., Tewksbury, MA, United States) and NaH^13^CO_3_ (0.176 mg/kg BW, 99%; Cambridge Isotope Laboratories, Inc.) to prime the bicarbonate pool and reduce the time to reach isotopic steady state (Shoveller et al., 2017; Pezzali et al., 2023). To maintain the supply of L-[1-^13^C]-Phe, the following seven meals contained constant doses L-[1-^13^C]-Phe (2.4 mg/kg BW) for the dog. Expired CO_2_ was collected over the last eight 25-min periods. Overall, during each IAAO study, the dog spent ∼6.3 h inside the oxidation chamber. A detailed timeline of the IAAO technique is outlined in Mansilla et al. (2018).

#### Sample collection and analysis

Breath samples and the expired CO_2_ within was collected according to Crosbie et al. (2024), using an open circuit calorimetry system. Fresh air was pulled into the oxidation chambers by a rotary vane vacuum pump through a series of DRIERITE-filled columns (calcium sulfate impregnated with colbalt chloride as an indicator; W. A. Hammond DRIERITE Co. Ltd) to the CO_2_ analyser (Qubit Model S155, Quibit Systems Inc., Kingston, ON) and the gas switcher. From the gas switcher, expired breath was pushed through midget bubbles, containing 8 mL of 1 mol/L sodium hydroxide (NaOH) solution. The NaOH solution was used to trap CO_2_ released by the dog while in the oxidation chambers for the subsequent ^13^CO_2_ enrichment analysis (Shoveller et al., 2017). Collected breath samples were then stored in an air-tight serum tube and kept at room temperature until further analysis. Calorimetry data was collected automatically using Qubit calorimetry software (Customized Gas Exchange System and Software for Animal Respirometry; Qubit Systems Inc.). Analysis of ^13^C enrichment in breath samples was conducted at the Environmental Isotope Laboratory at the University of Waterloo (Waterloo, ON, Canada). Breath samples were analyzed with a Gasbench II interfaced with a Delta V Plus mass spectrometer (Thermo Scientific, Bremen, Germany).

Ultraperformance liquid chromatography (UPLC) was used to measure free AA concentrations in plasma and whole blood to better understand the response to oxidation and any impacts of the test diets on macronutrient partitioning. To achieve this, 100 μL of plasma or whole blood was deproteinized using 100 μL of 10%-sulfosalicylic acid, vortexed, and then centrifuged at 14,000×g for 5 min. The supernatant was derivatized by an ACCQTag Ultra derivatization kit (Waters Corporation, Milford, MA, United States). Derivatized AA were separated in a column (2.1 mm × 100 mm, particle size: 1.7 μm) maintained at 55 °C with the use of UPLC (Waters Corporation) with UV detection (260 nm). AA peak areas were compared with known standards and analyzed with Waters Empower 2 Software (Waters Corporation). Concentrations of total plasma Cys, homocysteine (Hcys), and glutathione (GSH) were analyzed using UPLC, following an adapted method from Vester and Rasmussen (1991) and Pfeiffer et al. (1999), as detailed in Crosbie et al. (2024).

Nutrient contents of the BAS and ChM63 diets were analyzed for DM, crude protein, crude fat, crude fiber, total dietary fiber, soluble dietary fiber, insoluble dietary fiber, and all AA at the commercial laboratory Eurofins Microbiology Laboratories (Madison, Wisconsin, United States). AAs in the BAS and ChM63 diets, except for Trp, Met, cystine, and taurine (Tau) were analyzed using the acid hydrolysis procedure (AOAC, 2005; 982.30). Methionine and cystine were quantified in using the oxidative hydrolysis procedure (AOAC, 2005; method 994.12), Trp was analyzed using the alkaline hydrolysis procedure (AOAC, 2005; method 988.15), and Tau was analyzed using method 999.12 (AOAC, 2005).

### Experiment 2: Determining metabolic availability of methionine using the reformulated crystalline amino acid reference diet

All seven of the previously acquired neutered male mixed-breed hound dogs (3 yr old; 26.3 kg ± 2.0 kg BW) were used to complete this study. All seven dogs were housed, socialized, and acclimated to the oxidation chambers as indicated above.

#### Diets and study design

Three extruded kibble diets were formulated to determine the MA of Met in peas and ChM (Table 1). These included the same corn starch and barley-based BAS diet providing Met at 19% of its requirement for Labrador Retrievers (BAS19+: 0.10% DM, Table 3; Mansilla et al., 2020), a ChM and lamb-based diet (ChM63), and a chipped green pea and lamb-based diet (PEA63) both providing Met at 63% of its requirement (0.32 and 0.33% DM, respectively; Mansilla et al., 2020). All protein-containing ingredients were added to hit pre-determined Met content targets, but not crude protein contents, at the expense of corn starch (Table 3). As was reported in Crosbie et al. (2025), all diets were formulated to be isoenergetic (3500 kcal/kg as-fed). However, after formulation and production of test diets was complete, it was determined through calculating the ME of all test diets using the modified Atwater calculation, that the provision of ME in the PEA63 and ChM63 diets was 4.9 and 4.5% greater, respectively, than that of the ME provision in the BAS19 diet. We did not consider that this was a significant difference among diets. All other nutrients, apart from fiber, were formulated to be held constant across all three diets. Fiber was not held constant due to the inherently high fiber content of peas, since this is an intrinsic difference between animal-based and pulse-based ingredients (Silvio et al., 2000). Across all test diets, barley was provided at similar inclusion levels, with the BAS19 diet providing barley at an inclusion level of 25.85% (versus 25.0% in the ChM63 and PEA63 diets), in order to improve the extrusion of a primarily corn-starch-based diet. Inclusion levels of lamb meal and deboned lamb were the same in both the ChM63 and PEA63 diets (15 and 9.5% as-fed, respectively). Barley, deboned lamb, and lamb meal were used due to their inherently low Met content to allow for changes in the MA of Met to be reflective of the inclusion level of ChM and peas (Moehn et al., 2005; NRC, 2006). Inclusion levels of vitamins and minerals provided in the BAS19, ChM63, and PEA63 diets varied to provide all vitamins and minerals above the AAFCO recommendations for adult dogs at maintenance (AAFCO, 2023), and were based on the levels of vitamins and minerals provided by the primary ingredients (i.e., barley and ChM in the ChM63 diet, but only barley in the BAS19 diet). Extrusion processing parameters for all three diets were set to achieve a similar wet bulk density to ensure consistent cooking and to achieve similar specific mechanical energies (HP/hr/ton) off the extruder (BAS19: 95.3, ChM63: 97.8, PEA63: 97.8).

All IDAA in the BAS19, ChM63, and PEA63 diets were added as free AA using the methods outlined in Exp. 1 and then top-dressed on the diet before feeding as a proportion of the amount of diet fed. Phenylalanine, Tyr, and Cys content of all diets were supplemented to hit the same targets used in Exp. 1, to ensure Phe would be shunted to either protein synthesis or oxidation and not to synthesize Tyr and that sulfur AA metabolism would not be limited when Met was provided at 19% of its requirement in the BAS19+ diet (Table 3; Shiman and Gray, 1998). Tryptophan was provided to 120% of the AAFCO (2023) recommendation for adult dogs at maintenance on a DM basis in the ChM63 diet to ensure protein synthesis would not be limited by another AA, as this was the only IDAA that fell below this threshold. Supplementation of all other IDAA in the BAS reference diets were provided to match the ChM63 diet, which as determined in Exp. 1, had the greatest (ie most negative) oxidation response. Diets were made isonitrogenous via supplementation with free Ala in solution when appropriate and prepared using the same standards outlined in Exp. 1. Two additional BAS treatments were created by supplementing Met as DL-Met in solution to 40 and 63% of the requirement (BAS40**+**: 0.21% DM and BAS63+: 0.33% DM; Mansilla et al., 2020). This resulted in three graded levels of Met for the BAS reference diet to allow for a slope-ratio assay approach with the BAS19+ diet as the common first point (Moehn et al., 2005). We previously conducted a MA study using three graded levels of Met for both ChM and peas (Crosbie et al., 2025), which used the same experimental design, ChM63 and PEA63 diet batches, and supplemental AA formulations. Therefore, for this study, we chose to use only two graded levels of Met for both ChM and peas to act as a comparison to the BAS reference diet used in this study.

The study design was a partially replicated 5 × 5 Latin square (*n* = 7), where all dogs were randomly assigned to one of the five groups of dietary treatments in each experimental period, with no dog receiving the same order of treatments and ensuring that all treatments were represented on each calorimetry day per period. During the 14-d diet adaptation period, dogs were fed a commercial wash-in diet (S6 Nutram Sound Balanced Wellness Adult Dog Food, chicken meal and brown rice recipe; Elmira Pet Products, Elmira, ON, Canada) once daily at 0800 h in amounts known to maintain ideal individual BW based on historical feeding records. Dogs were allowed 15 min to finish their daily meal, and all dogs finished their daily ration during that time. There were five 3-d experimental periods conducted consecutively; the first 2 d were diet adaptation to the treatment diet, followed by breath collection on day 3. As outlined in Exp. 1, typical IAAO studies require feeding animals the same amount of food in g/kg BW during the 2-d diet adaptation period to ensure all dogs are adapted to receiving the same amount of dietary nitrogen (Moehn et al., 2005; Shoveller et al., 2017). However, despite all dogs in this study being of similar ideal BW, their daily energy requirement to maintain ideal BW varied from 1013 to 1714 kcal/d. Therefore, all dogs continued to be fed to maintain ideal BW during the 2-d diet adaptation period. Additionally, the same quantity of each experimental diet on an energy basis was fed within dog across all days of the study to control for this variation (Moehn et al., 2005; Shoveller et al., 2017). As was done in Exp. 1, all free-AA supplementation was dosed as a proportion of diet fed, to ensure that all dogs received the same proportion and balance of AA per total daily amount fed. On IAAO breath collection days, food intake was restricted to 13 g/kg BW, which varied between 70 and 100% of the historical feeding allowance known to maintain ideal BW, based on historical feeding records. After completion of each IAAO, dogs that had not received their total daily feed allowance received the remainder. This feeding protocol ensured that dogs consumed all the test diets during IAAO and received equivalent isotope delivery. This protocol was repeated five times until all dogs received all treatments. Blood samples (5 mL) were collected from each dog within 30 min of their final meal at the end of each IAAO day and were prepared and stored for analysis of fed-state plasma and whole blood AA, as described in Exp. 1.

#### Indicator amino acid oxidation study, sample collection, and analysis

Each IAAO study was conducted using the same methods outlined above in Exp. 1 for all dogs. All breath and blood samples, plasma and whole blood AA, and nutrient contents of the BAS, ChM63, and PEA63 test diets were collected and analyzed using the same methods as outlined in Exp. 1.

#### Calculations

The fraction of ^13^CO_2_ released per kg of BW per h (F^13^CO_2_ mmol/kg BW/h) was calculated using the following equation:


F13 CO2 (mmol/kg/h)=(VCO2) (APE) (44.6) (60)(BW) (1.0) (100)

In which VCO_2_ is the average production of CO_2_ from all breath collection days within each dog at resting in mL/min; APE (atom percent excess) is the average ^13^CO_2_ enrichment in expired breath at isotopic steady state in percent; and BW is the weight of the dog in kg. The constants 44.6 (mmol/mL) and 60 (min/h) convert the VCO_2_ to micromoles per hour; the factor 100 changes APE to a fraction; and the 1.0 is the retention factor of CO_2_ in the body due to bicarbonate fixation as reported previously (Shoveller et al., 2017).

The MA of Met in Exp. 2 was calculated following the equation by Littell et al. (1997):


$$\mathrm{Metabolic}\;\mathrm{availability}=\frac{bT}{bR}$$

where bT and bR were the slopes from the IAAO response (ie F^13^CO_2_) following graded intake of the pea test diets and reference CM diets, respectively.

Resting and fed energy expenditure (REE, FEE) were calculated based on VO_2_ and VCO_2_ using the modified Weir equation (Weir, 1949):


Energy expenditure (kcal/d) = 3.94(VO2)+1.11(VCO2)

In which VO_2_ and VCO_2_ are the volume of oxygen consumed by the dog and the volume of carbon dioxide produced (L/d) and energy expenditure (kcal/d) was expressed in relation to metabolic BW (BW^0.75^; mBW) for all dogs. Resting and fed respiratory quotient (RQ) was calculated directly by the Qubit calorimetry software as CO_2_ production/O_2_ production. Fed fat and carbohydrate oxidation (g/min/kg BW) were calculated using the equations established by Frayn (1983):


$$\mathrm{Carbohydrate}\;\mathrm{oxidation}\;(g/\min/\mathrm{kg}\;\mathrm{BW})\;=\;\frac{4.59(\mathrm{VCO}2)-3.22(VO2)}{BW}$$

$$\mathrm{Fat}\;\mathrm{oxidation}\;(g/\min/\mathrm{kg}\;\mathrm{BW})\;=\frac{1.70(\mathrm{VCO}2)-1.70(VO2)}{BW}$$

In which VCO_2_ and VO_2_ are the volume of carbon dioxide produced and the volume of oxygen consumed by the dog (L/min), respectively, and BW is the weight of the dog in kg.

### Statistical analysis

Statistical analyses were conducted using SAS (Version 9.3, SAS Institute Inc., Cary, NC, United States of America). Plasma and whole blood AA, BW, mBW, REE, FEE, fasted and fed RQ, fat oxidation, and carbohydrate oxidation were analyzed using PROC GLIMMIX, with diet as the fixed effect and dog and period as random effects. Data were expressed as least squares means ± SEM, and means were separated using the Tukey-Kramer post-hoc test when a statistical difference was observed. Due to differences in the number of completed treatments the denominator degrees of freedom were not constant across least squares means, therefore, variability in the denominator degrees of freedom was adjusted using the Games-Howell method to provide a more conservative interpretation of statistical significance (Games and Howell, 1976). Methionine intake was expressed as the % of Met from the estimated requirement determined by Mansilla et al. (2020) for Labrador Retrievers (0.52% DM), which were of similar size to the dogs used in the present study. Regression within the analysis of variance was determined using PROC GLIMMIX to construct the regression equation for each of the dietary treatments (BAS, ChM, and PEA test diets), to obtain the slopes of the three lines. The effect of Met inclusion, the addition of Met via ChM, pea, or crystalline AA inclusion, and their interactions on the variation of F^13^CO_2_, were tested using PROC GLIMMIX, with dog and period as random effects. This procedure also tested whether F^13^CO_2_ slopes were significantly different from zero. F^13^CO_2_ results were expressed as a regression equation. As the experimental design, diet formulation, sample collection, and analysis methods (calculation of F^13^CO_2_, plasma and whole blood AA, BW, mBW, REE, FEE, fasted and fed RQ, fat oxidation, and carbohydrate oxidation) were identical between Exp. 1 and 2 for the dog fed the BAS63+ diet, data were pooled with those of Exp. 2 and analyzed using the same statistical methods described above. Results were considered statistically significant *P *≤ 0.05.

## Results

### Experiment 1: Pilot trial to determine reformulation of a crystalline amino acid reference diet

The dog remained healthy and maintained ideal BW throughout the study. This dog completed one of the two experimental periods and consumed all meals on the IAAO sampling day for the BAS63+ diet. The dog did not consume all meals on the IAAO sampling day for the BAS63***−*** diet, and therefore did not reach isotopic steady state, which is required for IAAO MA studies (Moehn et al., 2005; Shoveller et al., 2017).

#### Oxidation response of the BAS63− and BAS63+ diets

Despite this dog not completing the experimental period testing the BAS63***−*** diet, this dog had previously completed an identical IAAO sampling day using the same experimental procedures and formulation of the BAS63***−*** diet (Crosbie et al., 2025). Therefore, this data was used as a comparison to the BAS63+ diet in this study. Results for the oxidation response (F^13^CO_2_) of the BAS63***−*** and BAS63+ diets in response to different IDAA compositions are presented in Table 4. The oxidation response was 84% less for the BAS63+ diet compared to the BAS63***−*** diet. Therefore, the BAS63+ diet crystalline AA formulation was used for Exp. 2.

**Table 4.** Rate of L-[1-^13^C]-Phenylalanine oxidation (F^13^CO_2_) in response to intake of two basal reference diets containing crystalline amino acids where; 1) all indispensable amino acids were provided at 120% of AAFCO recommendations for adult dogs at maintenance (BAS63−), and 2) all indispensable amino acids were provided to match the chicken diet (BAS63+) in one dog (n = 1) after indicator amino acid oxidation

	Diet	
	BAS63−^1^	BAS63+
**F^13^CO_2_, umol/kg/h**	4.1335	1.6803

1 Result for F^13^CO_2_ (umol/kg/h) for the BAS63− diet is from Crosbie et al. (2025) which used the same dog, diet composition, and experimental procedures as was used in this pilot study.

### Experiment 2: Determining metabolic availability of methionine using the reformulated crystalline amino acid reference diet

All dogs remained healthy and maintained their BW throughout the study. Two dogs completed all five experimental periods and consumed all meals on all IAAO sampling days. Five dogs did not complete certain treatments, as they did not consume all 13 meals during the IAAO breath collection day, and therefore, they did not reach isotopic steady state, which is required for IAAO MA studies (Moehn et al., 2005; Shoveller et al., 2017). The first of the remaining five dogs completed all treatments except for BAS40+. The second of the remaining five dogs completed all treatments except for BAS40+ and BAS63+. The third of the remaining five dogs completed all treatments except for BAS19+ and BAS40+. The fourth and fifth of the remaining five dogs only completed the ChM63 and PEA63 dietary treatments. Two dogs, one fed the ChM63 diet and one fed the PEA63 diet, consumed all meals for IAAO but did not reach isotopic steady state. This resulted in a total of six observations for the PEA63 and ChM63 diets, five observations for the BAS63+ diet (including the one observation from Exp 1), four observations for the BAS19+ diet, and two observations for the BAS40+ diet.

#### Metabolic availability of Met in peas and chicken meal

**Figure 1.**
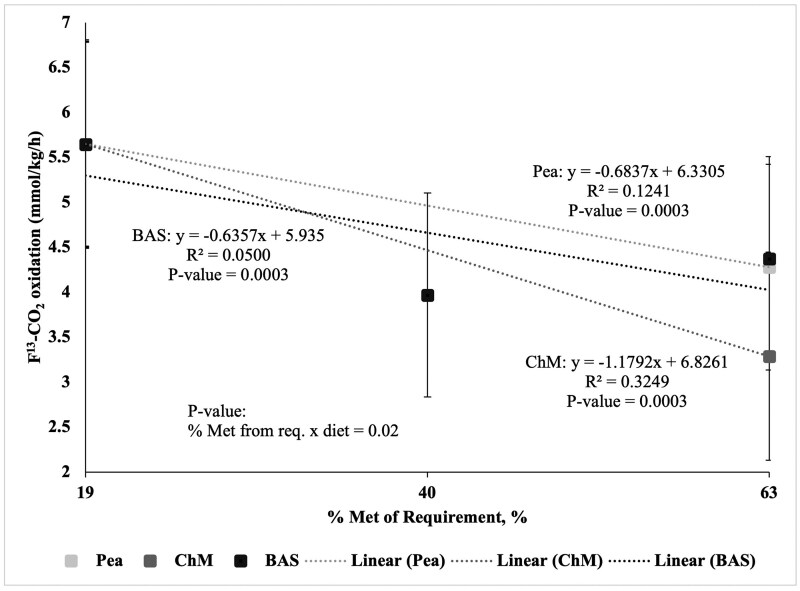
Linearity of the rate of L-[1-^13^C]-Phe oxidation (F^13^CO_2_) in response to graded intake of protein-bound Met from crystalline amino acids (BAS), chicken meal (ChM), and peas in adult dogs. Methionine from BAS was provided at 19 (*n* = 4), 40 (*n* = 2), and 63% (*n* = 5) of the requirement for Labrador Retrievers (0.52% DM), respectively. Methionine from both CM (*n* = 6) and peas (*n* = 6) was provided at 63% of the requirement for Labrador Retrievers (0.52% DM).

The increasing concentration of Met, the different diet types, and the interaction between the two were sources of variation for F^13^CO_2_ (*P *< 0.05; Figure 1). As Met intake from the crystalline AA reference diet (i.e., BAS19+, BAS40+, and BAS63+) increased from 19 to 63% of the requirement for Met (0.52% DM; Mansilla et al., 2020), the rate of ^13^C-Phe oxidation (F^13^CO_2_) decreased linearly. A negative slope of the best fit line of ***−***0.6357 ± 0.01 (*P *< 0.05; Figure 1) was determined using linear regression for the BAS crystalline AA reference protein. As Met from the common first point and ChM increased from 19 to 63% of the requirement for Met (0.52% DM; Mansilla et al., 2020), the rate of ^13^C-Phe oxidation (F^13^CO_2_) decreased linearly. A negative slope of the best fit line of -1.1792 ± 0.01 (*P *< 0.05; Figure 1) was determined using linear regression for ChM. As Met from the common first point and peas increased from 19 to 63% of the requirement of Met (0.52% DM; Mansilla et al., 2020), the rate of ^13^C-Phe oxidation (F^13^CO_2_) decreased linearly. A negative slope of the best fit line of ***−***0.6837 ± 0.01 (*P *< 0.05; Figure 1) was determined using linear regression for peas. However, as the negative slope of the rate of ^13^C-Phe oxidation (F^13^CO_2_) was greater (ie more negative) for both peas and ChM than the BAS crystalline AA reference protein, the MA of Met in both peas and ChM could not be calculated and determined. Despite this, the MA of Met in peas was 58% relative to ChM, based on the ratio of the slope of oxidation between the two.

#### Body weight and calorimetry data

Body weight and calorimetry data are presented in Table 5. There was no effect of diet for BW (*P *= 0.850), mBW (*P *= 0.842), REE (*P *= 0.582), and FEE (*P *= 0.247) across all dietary treatments, which is a requirement to assess the effect of dietary Met on protein synthesis without changes in whole body energy metabolism. There was an effect of diet on fasted RQ, where dogs fed the BAS19+ diet had greater fasted RQ than those fed the ChM63 and PEA63 diets (*P *< 0.001). Additionally, dogs fed the BAS40+ and BAS63+ diet had greater fasted RQ than those fed the PEA63 diet (*P *< 0.001). There was an effect of diet on fed RQ, where dogs fed the BAS19+, BAS40+, and BAS63+ diets had greater fed RQ than those fed the PEA63 diet (*P *< 0.001). Fat oxidation was greater in dogs fed the ChM63 and PEA63 diets than those fed the BAS19+. BAS40+, and BAS63+ diet (*P *< 0.001). Dogs fed the ChM63, BAS19+, BAS40+, and BAS63+ diets had greater carbohydrate oxidation than those fed the PEA63 diet (*P *< 0.05).

**Table 5.** Bodyweight (BW, kg and BW^0.75^, kg) and indirect calorimetry data for all adult large mixed-breed dogs used after indicator amino acid oxidation presented as least square means ± SEM

	Diet^1^					
Item	BAS19+	BAS40+	BAS63+	ChM63	PEA63	*P*-value
***n***	4	2	5	6	6	
**% Met req.**	19	40	63	63	63	
**BW, kg**	26.2 ± 0.91	26.1 ± 0.94	26.4 ± 0.91	26.4 ± 0.90	26.2 ± 0.90	0.850
**mBW, kg^2^**	11.6 ± 0.30	11.6 ± 0.31	11.6 ± 0.30	11.6 ± 0.30	11.6 ± 0.30	0.842
**Resting EE, kcal/kg^0.75^**	95.1 ± 4.31	98.1 ± 5.64	95.0 ± 4.10	89.7 ± 3.96	91.5 ± 3.72	0.582
**Fed EE, kcal/kg^0.75^**	101.9 ± 3.33	94.7 ± 4.42	98.2 ± 3.16	105.0 ± 3.03	100.3 ± 2.83	0.247
**Fasted RQ**	0.907 ± 0.021^a^	0.888 ± 0.025^a, b^	0.877 ± 0.017^a, b^	0.817 ± 0.015^b, c^	0.783 ± 0.015^c^	<0.001
**Fed RQ**	0.889 ± 0.014^a^	0.891 ± 0.017^a^	0.873 ± 0.011^a^	0.837 ± 0.010^a, b^	0.802 ± 0.010^b^	<0.001
**Fat oxidation, g/min/kg BW**	0.068 ± 0.009^b^	0.062 ± 0.012^b^	0.077 ± 0.007^b^	0.108 ± 0.006^a^	0.126 ± 0.006^a^	<0.001
**Carbohydrate oxidation, g/min/kg BW**	0.319 ± 0.026^a^	0.305 ± 0.031^a^	0.288 ± 0.022^a^	0.257 ± 0.019^a^	0.180 ± 0.019^b^	0.002

1 BAS19+ = Corn starch and barley-based reference diet containing crystalline AA providing Met at 19% of the requirement; BAS40+ = Corn starch and barley-based reference diet containing crystalline AA providing Met at 40% of the requirement; BAS63+ = Corn starch and barley-based reference diet containing crystalline AA providing Met at 63% of the requirement; ChM63 = Chicken diet containing chicken and lamb as the primary protein sources providing Met at 63% of the requirement; PEA63 = Pea diet containing peas and lamb as the primary protein sources providing Met at 63% of the requirement.

2 mBW = BW^0.75.

#### Plasma and whole blood AA

The results for plasma and whole blood AA from blood samples taken after each IAAO sampling day are presented in Table 6. There was an effect of diet for most of the analyzed AA that generally followed one of two patterns; 1) AA provided in the greatest amounts as free-AA typically resulted in greater concentrations of those AA in plasma and whole blood (i.e., in the BAS19+, BAS40+, and BAS63+ diets) and 2) AA that were provided in the greater amounts than in other dietary treatments typically resulted in greater concentrations of the AA in plasma and whole blood (i.e., the PEA63 diet followed by the ChM63, BAS63+, BAS40+, and BAS19+ diets).

**Table 6.** Fed plasma and whole blood amino acid concentrations (lsmeans ± SEM nmol/mL) after indicator amino acid oxidation in adult large mixed-breed dogs

	Diet^1^					
Item	BAS19+	BAS40+	BAS63+	ChM63	PEA63	*P*-value
***n***	4	2	5	6	7	
**% Met req.**	19	40	63	63	63	
**Cys**	104.0 ± 4.42^b^	105.4 ± 5.64^b^	106.5 ± 4.22^b^	124.5 ± 3.87^a^	134.7 ± 3.64^a^	<0.001
**Hcys**	6.0 ± 0.98^b^	5.2 ± 1.37^b^	6.8 ± 0.97^a, b^	7.6 ± 0.84^a, b^	9.4 ± 0.77^a^	0.026
**GSH**	10.8 ± 1.10	9.5 ± 1.62	10.9 ± 1.10	10.0 ± 0.93	8.8 ± 0.90	0.497
**Plasma AA**						
***Indispensable***						
**Arg**	138.4 ± 14.52^b^	158.6 ± 20.61^a, b^	130.6 ± 14.48^b^	142.4 ± 12.88^b^	208.2 ± 11.90^a^	<0.001
**His**	88.7 ± 3.00	101.1 ± 4.00	98.2 ± 2.83	94.2 ± 2.53	96.5 ± 2.35	0.053
**Ile**	81.5 ± 10.22	103.7 ± 14.35	72.7 ± 10.20	63.7 ± 8.89	86.7 ± 8.15	0.067
**Leu**	160.8 ± 11.18^a, b^	185.2 ± 15.68^a^	149.1 ± 11.13^a, b, c^	111.3 ± 9.62^c^	128.7 ± 8.79^b, c^	0.003
**Lys**	171.7 ± 13.01^a^	184.1 ± 16.19^a^	133.9 ± 13.43^b^	130.1 ± 12.05^b^	171.3 ± 11.46^a^	<0.001
**Met**	21.4 ± 2.50^d^	47.6 ± 3.66^b^	64.2 ± 2.65^a^	42.7 ± 2.09^b^	33.7 ± 1.89^c^	<0.001
**Phe**	102.0 ± 8.99	109.8 ± 12.21	96.2 ± 9.02	95.9 ± 7.91	91.4 ± 7.31	0.587
**Thr**	217.3 ± 18.91^a^	176.8 ± 27.36^a, b^	151.3 ± 19.85^a, b^	130.0 ± 16.42^b^	145.4 ± 15.00^b^	0.012
**Trp**	114.1 ± 7.44^a, b^	110.3 ± 9.78^a, b^	105.3 ± 7.06^b^	126.3 ± 6.37^a, b^	128.9 ± 5.94^a^	0.036
**Val**	228.6 ± 9.23^a^	246.0 ± 11.07^a^	216.1 ± 8.40^a, b^	193.1 ± 7.32^b^	199.3 ± 6.72^b^	0.005
***Dispensable***						
**Ala**	1226.4 ± 70.59^b^	1638.2 ± 101.14^a^	1271.2 ± 69.94^b^	784.6 ± 61.97^c^	419.1 ± 55.73^d^	<0.001
**Asn**	56.8 ± 4.04^b^	60.1 ± 5.15^b^	54.2 ± 3.85^b^	65.2 ± 3.53^b^	97.6 ± 3.73^a^	<0.001
**Asp**	10.1 ± 0.92	9.7 ± 1.25	10.2 ± 0.93	9.7 ± 0.82	7.7 ± 0.76	0.053
**Cystine**	7.2 ± 1.34^c^	7.5 ± 1.77^b, c^	9.2 ± 1.35^b, c^	12.5 ± 1.23^a, b^	13.1 ± 1.17^a^	0.002
**Tyr**	118.4 ± 10.06^a, b^	124.6 ± 13.59^a, b^	100.5 ± 10.24^a, b^	117.9 ± 9.12^a^	90.6 ± 8.53^b^	0.023
**Glu**	56.7 ± 3.40	56.0 ± 4.55	52.2 ± 3.54	59.0 ± 3.05	57.8 ± 2.83	0.444
**Gly**	159.8 ± 18.23^b^	175.6 ± 25.24^b^	167.1 ± 18.04^b^	297.5 ± 17.36^a^	283.2 ± 15.35^a^	<0.001
**Pro**	143.1 ± 10.52^c^	168.4 ± 13.94^c^	146.3 ± 10.69^c^	275.2 ± 9.70^a^	227.3 ± 9.76^b^	<0.001
**Ser**	117.5 ± 12.01	123.3 ± 15.78	122.1 ± 12.12	145.3 ± 11.38	127.6 ± 10.08	0.264
**Tau**	61.6 ± 9.72	68.4 ± 12.75	64.2 ± 9.80	81.2 ± 8.70	87.5 ± 8.12	0.081
***Whole blood***						
**Indispensable**						
**Arg**	225.9 ± 10.75^b^	224.2 ± 13.15^b^	207.0 ± 10.87^b^	223.9 ± 10.12^b^	275.6 ± 9.87^a^	<0.001
**His**	110.9 ± 4.67	112.4 ± 6.41	112.1 ± 4.73	117.5 ± 4.08	119.8 ± 3.72	0.409
**Ile**	93.8 ± 5.75	103.1 ± 8.13	86.1 ± 5.14	84.2 ± 4.69	92.4 ± 4.34	0.286
**Leu**	182.5 ± 9.10^a^	189.7 ± 13.12^a^	168.6 ± 8.39^a, b^	144.5 ± 7.30^b^	150.6 ± 6.72^a, b^	0.010
**Lys**	338.2 ± 10.63^a^	311.4 ± 13.00^a, b, c^	280.4 ± 10.63^b, c^	276.4 ± 9.60^c^	301.1 ± 9.60^b^	<0.001
**Met**	28.5 ± 4.46^b^	32.8 ± 7.06^a, b^	53.2 ± 5.09^a^	43.4 ± 4.05^a, b^	36.4 ± 3.39^a, b^	0.006
**Phe**	103.2 ± 6.28	101.6 ± 8.40	94.2 ± 5.92	101.0 ± 5.29	91.1 ± 4.91	0.305
**Thr**	245.4 ± 12.17^a^	203.8 ± 17.05^a, b^	185.7 ± 12.82^b^	155.3 ± 11.56^b^	173.1 ± 10.05^b^	<0.001
**Trp**	50.6 ± 2.66^a, b^	46.1 ± 3.49^a, b^	45.6 ± 2.51^b^	53.0 ± 2.65^a, b^	54.3 ± 2.10^a^	0.042
**Val**	238.8 ± 6.12^a^	240.3 ± 7.45^a^	220.9 ± 5.26^a, b^	203.3 ± 4.74^b^	211.7 ± 4.31^b^	<0.001
**Dispensable**						
**Ala**	1128.3 ± 58.46^a^	1367.3 ± 82.98^a^	1140.8 ± 58.67^a^	714.8 ± 50.42^b^	417.3 ± 45.77^c^	<0.001
**Asn**	42.4 ± 4.76^b^	43.2 ± 5.47^b^	39.3 ± 4.65^b^	46.1 ± 4.46^b^	56.5 ± 4.34^a^	<0.001
**Asp**	62.8 ± 4.36	66.9 ± 5.60	58.8 ± 4.33	58.4 ± 3.91	64.6 ± 3.69	0.230
**Cystine**	1.0 ± 0.30	2.2 ± 0.44	1.2 ± 0.30	1.0 ± 0.27	1.2 ± 0.24	0.212
**Tyr**	130.8 ± 7.13^a^	117.1 ± 9.11^a, b, c^	109.4 ± 7.19^b, c^	123.0 ± 6.64^a, b^	107.4 ± 6.34^c^	0.005
**Glu**	188.8 ± 11.14	191.0 ± 14.77	193.4 ± 11.25	203.0 ± 10.17	215.2 ± 9.59	0.116
**Gly**	211.4 ± 12.80^b^	207.6 ± 17.82^b^	197.8 ± 12.78^b^	331.3 ± 11.12^a^	298.9 ± 10.73^a^	<0.001
**Pro**	178.6 ± 8.96^c^	187.5 ± 11.55^c^	165.2 ± 9.09^c^	267.7 ± 8.29^a^	243.1 ± 8.01^b^	<0.001
**Ser**	183.6 ± 11.65	187.8 ± 15.52	169.4 ± 11.68	198.2 ± 10.87	184.6 ± 9.52	0.304
**Tau**	149.8 ± 11.15	155.9 ± 14.80	141.7 ± 10.98	165.2 ± 9.76	171.3 ± 9.09	0.127

1 BAS19+ = Corn starch and barley-based reference diet containing crystalline AA providing Met at 19% of the requirement; BAS40+ = Corn starch and barley-based reference diet containing crystalline AA providing Met at 40% of the requirement; BAS63+ = Corn starch and barley-based reference diet containing crystalline AA providing Met at 63% of the requirement; ChM63 = Chicken diet containing chicken and lamb as the primary protein sources providing Met at 63% of the requirement; PEA63 = Pea diet containing peas and lamb as the primary protein sources providing Met at 63% of the requirement.

For plasma AA, Cys concentrations were greater in the ChM63 and PEA63 diets than the BAS19+, BAS40+, and BAS63+ diets (*P *< 0.001) and Hcys concentrations were greater in the PEA63 diet compared to the BAS19+ and BAS40+ diets (*P *< 0.05). Of the plasma IDAA, arginine (Arg) concentrations were greater in the PEA63 diet than the BAS19+, BAS63+, and ChM63 diets (*P *< 0.001). Leucine (Leu) concentrations were greater in the BAS40+ diet than the ChM63 and PEA63 diets (*P *< 0.05) and greater in the BAS19+ diet than the ChM63 diet (*P *< 0.05). Plasma lysine (Lys) concentrations were greater in the BAS19+, BAS40+, and PEA63 diets than the BAS63+ and ChM63 diets (*P *< 0.001). Methionine concentrations were greatest in the BAS63+ diet, followed by the BAS40+ and ChM63 diets, the PEA63 diet, and the BAS19+ diet (*P *< 0.001). Concentrations of threonine (Thr) were greater in the BAS19+ diet than both the ChM63 and PEA63 diets (*P *< 0.05) and Trp concentrations were greater in the PEA63 diet compared to the BAS63+ diet (*P *< 0.05). Plasma valine (Val) concentrations were greater in the BAS19+ and BAS40+ diets than the ChM63 and PEA63 diets (*P *< 0.05). Of the plasma dispensable AA, Ala concentrations were greatest in the BAS40+ diet followed by the BAS19+ and BAS63+ diets, the ChM63 diet, and finally the PEA63 diet (*P *< 0.001). Asparagine (Asn) concentrations were greater in the PEA63 diet compared to all other treatments (*P *< 0.001). Concentrations of cystine were greater in the PEA63 diet than the BAS19+, BAS40+, and BAS63+ diets (*P *< 0.05) and greater in the ChM63 diet than the BAS19+ diet (*P *< 0.05). Plasma Tyr concentrations were greater in the ChM63 diet than the PEA63 diet (*P *< 0.05) and glycine (Gly) concentrations were greater in the ChM63 and PEA63 diets than the BAS19+, BAS40+, and BAS63+ diets (*P *< 0.001). Concentrations of proline (Pro) were greatest in the ChM63 diet followed by the PEA63 diet, and the BAS19+, BAS40+, and BAS63+ diets (*P *< 0.001). There was no effect of diet observed for concentrations of plasma GSH (*P *= 0.497), histidine (His; *P *= 0.053), isoleucine (Ile; *P *= 0.067), Phe (*P *= 0.587), aspartate (Asp; *P *= 0.053), glutamate (Glu; *P *= 0.444), serine (Ser; *P *= 0.264), and Tau (*P *= 0.081) across all dietary treatments.

For the whole blood IDAA, there was an effect of diet for Arg (*P *< 0.001), where concentrations were greater in the PEA63 diet than all other dietary treatments. Whole blood Leu concentrations were greater in the BAS19+ and BAS40+ diets than the ChM63 diet (*P *< 0.05) and Lys concentrations were greatest in the BAS19+ diet followed by the ChM63 diet, and then the PEA63 diet (*P *< 0.001). Methionine concentrations were greater in the BAS63+ diet than the BAS19+ diet (*P *< 0.05) and Thr concentrations were greater in the BAS19+ diet than the BAS63+, ChM63, and PEA63 diets (*P *< 0.001). Concentrations of Trp were greater in the PEA63 diet than the BAS63+ (*P *< 0.05) and Val concentrations were greater in the BAS19+ and BAS40+ diets than the PEA63 and ChM63 diets (*P *< 0.001). Of the whole blood dispensable AA, Ala concentrations were greatest in the BAS19+, BAS40+, and BAS63+ diets, followed by the ChM63 diet, then the PEA63 diet (*P *< 0.001). Concentrations of Asn were greater in the PEA63 diet than any other dietary treatment (*P *< 0.001) and Tyr concentrations were greater in the BAS19+ diet than the BAS63+ and PEA63 diets (*P *< 0.05). Whole blood Gly concentrations were greater in the ChM63 and PEA63 diets than the BAS19+, BAS40+, and BAS63+ diets (*P *< 0.001). Proline concentrations were greatest in the ChM63 diet followed by the PEA63 diet, and the BAS19+, BAS40+, and BAS63+ diets (*P *< 0.001). There was no effect of diet observed for concentrations of whole blood His (*P *> 0.05), Ile (*P *> 0.05), Phe (*P *> 0.05), Asp (*P *> 0.05), cystine (*P *> 0.05), Glu (*P *> 0.05), Ser (*P *> 0.05), and Tau (*P *> 0.05) across all dietary treatments.

## Discussion

The first aim of this study was to determine if reformulating the IDAA composition of a semi-synthetic reference diet containing an extruded kibble base and crystalline AA would result in an improved oxidation response using the IAAO technique in adult dogs. Previous work in our laboratory (Crosbie et al., 2025) using an extruded reference diet where all IDAA, except for Met, were provided to at least 120% of the AAFCO recommendations for adult dogs at maintenance did not produce an oxidation response (AAFCO, 2023). In this previous work (Crosbie et al., 2025), the ChM63 diet produced the greatest (ie, most negative) oxidation response, therefore, in this study we chose to match the IDAA provision to the ChM63 diet (which was also used in this study). Specifically, arginine was increased from 0.38% to 0.88% (+132%), histidine from 0.13% to 0.24% (+85%), isoleucine from 0.28% to 0.41% (+46%), leucine from 0.48% to 0.84% (+75%), lysine from 0.59% to 0.69% (+17%), threonine from 0.41% to 0.46% (+12%), and valine from 0.35% to 0.54% (+54%). Using one dog, this resulted in an 84% improvement in the oxidation response between the previously used BAS63− diet and the new BAS63+ diet. The second aim of this study was to see if the new BAS63+ reference diet, with the updated IDAA profile, would result in an improved oxidation response when Met was provided at three graded levels (19%, 43%, and 63% of the Met requirement of Labrador Retrievers, respectively; Mansilla et al., 2020) and allow us to determine the MA of Met in both ChM and peas.

Despite the improved oxidation response observed in Exp 1, and the oxidation response of the reformulated BAS reference protein being improved by 179% compared to our previous BAS formulation (Crosbie et al., 2025), the oxidation response of the BAS diet was 7% and 60% less than peas and ChM, respectively. To determine the MA of AA in ingredients, the reference protein must have a lower (i.e., more negative) level of oxidation of the isotopic tracer (i.e., C^13^-Phe) to calculate AA MA (Littell et al., 1997; Moehn et al., 2005). As such, we could not determine the MA of Met using the new BAS diet formulation, ChM had the greatest oxidation response, and the MA of Met in ChM was 42% greater than peas. Additionally, the slopes of oxidation for ChM and peas used in this study were only 5% and 8% different from what we reported in our previous study, which used the same ingredient batches, diet processing, and AA provisions (Crosbie et al., 2025). This indicates that the IAAO technique for determining bioavailability of AA in ingredients produces repeatable results for ingredients from the same batch with the same AA provision.

It is unknown why the provision of free crystalline AA in the BAS diets did not create a comparable oxidation response to ChM, despite having the same IDAA provision. This could be due to several factors. Free AA are known to be rapidly absorbed into systemic circulation compared to protein-bound AA (Boirie et al., 1996; Weijzen et al., 2022). Weijzen et al. (2022) found no differences in mixed muscle protein synthesis rates when free AA were fed compared to protein-bound AA from intact milk protein in humans. It should be noted that the free AA mixture used in Weijzen et al. (2022) was comprised of only free AA, while in the present study the animal-protein-free extruded BAS diet did provide some protein-bound AA which were counted towards the total AA provision in the BAS19+, BAS40+, and BAS63+ diets. The amount of protein-bound AA in the BAS diet was on average 70% less than what was provided in the ChM63 diet, and it is unknown how a diet providing a mixture of protein-bound and free AA may have impacted protein synthesis in dogs, which likely differ from other animal models where the reference protein approach to the determination of MA has been used. Furthermore, the form in which dietary AA are provided can affect postprandial kinetics and metabolic fate of absorbed AA. Free AA typically result in a rapid and transient increase in plasma AA concentrations, leading to enhanced insulin response and increased AA catabolism in the liver (Eugenio et al. 2022). While this may stimulate protein synthesis, it may also reduce the efficiency of AA utilization due to saturation of anabolic pathways and increased oxidation. Conversely, intact proteins such as those found in ChM are digested more slowly, potentially leading to more gradual AA availability, sustained anabolic signaling, and reduced catabolism. It remains unclear how the mixed provision of free and protein-bound AA in the BAS diets may have modulated these postprandial responses. Eugenio et al. (2022) note that the optimal ratio of free to intact protein sources for maximizing protein retention and minimizing catabolism remains unknown in monogastric animals. As such, it is plausible that the lower content of intact protein in the BAS diets contributed to altered AA kinetics and reduced oxidation compared to the more protein-bound ChM diet, despite matched IDAA levels. Additionally, it is well established that there is a dietary requirement for 10 AA (Arg, His, Ile, Leu, Lys, Met, Phe, Thr, Trp, and Val), as these AA cannot be synthesized by the animal in adequate amounts, and thus, are classified as dietary indispensable (NRC, 2006). All other AA, including Ala, Asn, Asp, Cys, Glu, glutamine (Gln), Gly, Pro, Ser, and Tyr, are considered dispensable as they can be synthesized endogenously by the animal (NRC, 2006). While there is no established requirement for dispensable AA, some dispensable AA, particularly Gln and Gly, can stimulate protein synthesis via activating the mechanistic target of rapamycin (mTOR) pathway while also inhibiting protein breakdown (Wu, 2013). The total amount of dispensable AA provided in the ChM and BAS diets were similar after supplementation; however, Ala made up 78% of the total dispensable AA provision in the BAS+ diets, compared to only 41% in the ChM63 diet. Further, while the Gln content in the BAS and ChM63 diets was not determined analytically, the BAS diets provided 86% less Gly than the ChM63 diet. Therefore, it is possible that Gly was limiting in the BAS63+, BAS40+, and BAS19+ diets, which may have limited protein synthesis. Future studies should explore how providing a more balanced complement of dispensable AA in a reference diet may impact the oxidation response and support protein synthesis.

Concentrations of plasma Met in all diets, except for the BAS63+ diet, were below reported values for healthy adult dogs, which was expected as Met was provided below the requirement in all diets (Delaney et al., 2003). Identical levels of Met were provided in the ChM63, PEA63, and BAS63+ diets. Therefore, the greater concentration of Met in plasma in the BAS63+ diet indicates that the provision of Met as free-AA was more bioavailable and readily absorbed into circulation (Chung and Baker, 1992; NRC, 2006; Mansilla et al., 2020; Weijzen et al., 2022). This finding aligns with results observed by Crosbie et al. (2025), where equivalent levels of Met from the same ingredient sources resulted in similar outcomes. Whole blood Met concentrations were also elevated in the BAS63+ diet, but to a lesser extent. However, greater plasma Met from the BAS63+ diet did not result in greater plasma Cys compared to both the ChM63 and PEA63 diets. While the inclusion level of other compounds required to sustain methylation status, such as choline, folate, and vitamin B12, varied across diets, their concentrations were above the recommendations of adult dogs at maintenance (AAFCO, 2023). Cysteine was provided in equal amounts across all diets, albeit from different sources, and no differences were detected in other downstream sulfur AA metabolites, including plasma Hcys, GSH, and whole blood Tau. The lower Cys in the BAS63+ diet could be due to its lower dietary Gly content. Limited Gly could limit protein, therefore promoting greater Cys oxidation relative to the ChM63 and PEA63 diets. When protein synthesis is limited, greater release of AA into systemic circulation can occur, and may explain the elevated plasma Met with the BAS63+ diet (Shoveller et al., 2022).

Although we found many differences in other plasma and whole blood AA concentrations between dietary treatments, they typically mirrored the variations in the provision of those AA in the test diets, where the PEA63 diet contained the highest concentrations of most AA, followed by the ChM63, BAS63+, BAS40+, and BAS19+ diets. Despite this, when AA were supplemented as free crystalline AA in large quantities, these were generally found in greater concentrations in both plasma and whole blood (e.g., nearly 70% of the Val provision in the BAS63+, BAS40+, and BAS19+ diets was free Val compared to no supplementation in the ChM63 diet). This is expected as crystalline AA are estimated to be 100% bioavailable and readily absorbed into systemic circulation (Chung and Baker, 1992). It is important to note that apart from ensuring Met was provided by both CM, peas, and crystalline AA at precise graded levels below the requirement, the provisions of Cys, Phe, and Tyr provisions were standardized across all diets. There was no set upper limit of the provision of all other dietary AA in the ChM63 and PEA63 diets, only that they were supplied at or above 120% of the AAFCO (2023) recommendations for adult dogs at maintenance. However, the provision of Gly in the BAS19+, BAS40+, and BAS63+ diets was 150% less than both the ChM63 and PEA63 diets. This resulted in plasma Gly in the BAS19+, BAS40+, and BAS63+ diets being on average 56% and 52% less than the ChM63 and PEA63 diets, respectively, and 46% less than the reported concentrations of plasma Gly published by Delaney et al. (2003). Additionally, a similar pattern was observed between dietary provision and plasma AA concentrations for plasma Pro. Despite this, overall, concentrations of plasma and whole blood AA across dietary treatments were generally within reported ranges found in healthy adult dogs at maintenance and in agreement with previous findings from two similar IAAO MA study using the same population of dogs (apart from Ala, Phe, and Tyr, and Met which were above and below the normal range, respectively; Delaney et al., 2003; NRC, 2006; Beloshapka et al., 2018; Banton et al., 2021; Crosbie et al., 2024; Crosbie et al., 2025).

Body weight and energy expenditure in both fasted and fed states were similar across all treatments, suggesting that variations in dietary Met intake from crystalline AA, ChM, and peas did not affect energy metabolism (Moehn et al., 2005; Shoveller et al., 2017). Additionally, all dogs were fed to their individual energy requirements, as is required for design principles in MA studies on animals at maintenance (Moehn et al., 2005; Shoveller et al., 2017). Dogs fed the PEA63 diets had lower fasted RQ compared to the BAS19+ diet, lower fed RQ compared to the dogs fed any of the BAS diets, and lower carbohydrate oxidation compared to dogs fed all other diets. Additionally, dogs fed the ChM63 diet had lower fasted RQ compared to those fed the BAS19+ diet and both the PEA63 and ChM63 diets had greater fat oxidation compared to dogs fed any of the BAS treatments. This is likely due to several factors. Crosbie et al. (2025) used the same BAS, ChM63, and PEA63 diet compositions, albeit with different IDAA provisions in the BAS diets, in this study. In both studies, the BAS diets contained 14% more crude fat and nearly 18% more calculated nitrogen-free extract (NFE) than the PEA63 diet. Additionally, 18% of the total crude protein content in the BAS diets was derived from crystalline AA, compared to the intact proteins in the PEA63 diet. As also observed by Crosbie et al. (2025), it is probable that the substantial inclusion of highly bioavailable AA, combined with the higher NFE, led to a higher fasted and fed RQ (Goldenshluger et al., 2021). Furthermore, Yang et al. (1986) found that increased carbohydrate intake led to increased *de novo* Ala synthesis in humans. Therefore, it’s likely that both the increased proportion of carbohydrates and high level of Ala supplementation, particularly in the BAS diets (at nearly 13% DM) to make all diets isonitrogenous, led to a further increase in glycolysis and overall carbohydrate oxidation in these diets (Felig, 1973; Yang et al., 1986; Crosbie et al., 2025). Greater fat oxidation in the ChM63 diet compared to the BAS diets, and greater carbohydrate oxidation in the ChM63 diet compared to the PEA63 diets was also observed by Crosbie et al. (2025), which as mentioned previously, used similar diet compositions and crystalline AA provisions in the BAS, ChM63, and PEA63 diets as were used in this study, so was expected. Determining the MA of limiting AA in ingredients requires precise diet design to ensure diets are isoenergetic, isonitrogenous, and that key dietary AA are accurately provided. However, other macronutrient components, such as fiber, or overall macronutrient balance, are considered inherent to the ingredients themselves (Moehn et al., 2005). Therefore, it is reasonable that the different macronutrient compositions of the test diets and supplementation of Ala resulted in a shift in macronutrient oxidation.

The main limitations of this study were that only one dog was used in Exp. 1, with no replicates due to resource constraints, and the small sample size of only two observations for the BAS40+ diet in Exp. 2. Both limitations should be considered when interpreting these results. Palatability issues with the BAS diets, particularly the BAS40+ treatment, contributed to the reduced sample size. To account for this, adjustments to the denominator degrees of freedom were made during statistical analysis to provide a more conservative interpretation of statistical significance. Expectedly, this resulted in a greater standard error of the means for all parameters measured for this treatment. Despite this, the values obtained for the BAS40+ diet were generally similar to both the BAS19+ and BAS63+ treatments, which provided the same amount of AA, aside from Met, which had four and five observations each, respectively. It has been established that a sample size of four is suitable for conducting IAAO AA requirement studies in dogs (Mansilla et al., 2018; Templeman et al., 2019; Mansilla et al., 2020). Still, the results for the BAS40+ diet should be treated with caution, and future research should seek to improve the palatability of the semi-synthetic crystalline AA reference diet.

## Conclusions and Implications

The data presented above suggests that the diet design and execution of the IAAO technique to determine MA of Met in both ChM and peas was successful in creating a linear response in oxidation to two graded intakes of protein-bound Met from these ingredients. Additionally, the slopes of oxidation for both ChM and peas were similar to previous results, indicating the IAAO technique produces repeatable results when ingredients are processed identically. The oxidation response in the BAS diet was improved when the provision of IDAA AA was matched to ChM. However, the slope of oxidation from the BAS reference diet when the study was repeated with three graded levels of Met was greater than that of peas and ChM. Thus, the MA of Met peas could not be determined, but was less than ChM. The provision of IDAA from the BAS crystalline AA reference diet was identical to that provided by ChM and diets were isonitrogenous. However, both diets provided different proportions of dispensable AA. To define an acceptable reference protein formulation, future research should be done to identify the ideal dispensable AA composition in the BAS crystalline AA diet. Overall, further refinement of the reference diet is required to use this method to measure the MA of AA in ingredients fed to dogs.

## Abbreviations:

AA: amino acid
AAFCO: Association of American Feed Control Officials
APE: atom percent excess
BW: body weight
ChM: chicken meal
DM: dry matter
FEE: fed energy expenditure
IAAO: indicator amino acid oxidation
IDAA: indispensable amino acids
MA: metabolic availability
mBW: metabolic BW
ME: metabolizable energy
mTOR: mechanistic target of rapamycin
NaOH: sodium hydroxide
NFE: nitrogen-free extract
NRC: National Research Council
REE: resting energy expenditure
RQ: respiratory quotient
UPLC: ultra-performance liquid chromatography
VCO_2_: volume of expired carbon dioxide
VO_2_: volume of expired oxygen
